# Supplementary material for: Emergence and control of photonic band structure in stacked OLED microcavities
Source: Nat Commun. 2021 Oct 20;12:6111. doi: 10.1038/s41467-021-26440-3 (PMC8528838; doi:10.1038/s41467-021-26440-3)
Supplement: Supplementary file 1 — Supplementary Information [file 41467_2021_26440_MOESM1_ESM.pdf]

Supplementary Information: Emergence and Control of Photonic Band Structure in Stacked OLED Microcavities

David Allemeier[1], Benjamin Isenhart[2], Ekraj Dahal[1], Yuki Tsuda[3], Tsukasa Yoshida[4], and Matthew S. White[1,2,*]

[1]University of Vermont, Materials Science Program, Burlington VT, 05405, USA
[2]University of Vermont, Department of Physics, Burlington VT, 05405, USA
[3]Yamagata University, Graduate School of Science and Engineering, Yonezawa, Yamagata 992-8510, Japan
[4]Yamagata University, Graduate School of Organic Materials Science, Yonezawa, Yamagata 992-8510, Japan
[*]mwhite25@uvm.edu

Computational Modelling

The inherent complexity of designing and fabricating multi-layer OLED microcavity stacks requires a computational model to rapidly prototype devices and explore functionality. A Matlab program was developed to simulate the emission from the devices which implements the transfer matrix method of source terms of Benisty et al.[1] The program calculates the outcoupling strength at a given wavelength and external viewing angle using the wavelength-dependent complex index of refraction for each material and the dimensions of the stack. Additionally, the program accounts for surface roughness[2], exciton distribution profile[3], dipole orientation distribution and polarization components. This program is available at devphys.w3.uvm.edu/index/downloads, and further details of the theory are provided in the accompanying SI of Dahal et al.[4]

The simulations in this paper used ten dipole layer positions evenly distributed within each active layer to calculate the final outcoupling efficiency. The contributions were weighted by an exponential exciton distribution profile with a diffusion length of 3 nm.[3] The electron mobility of Alq_3 is greater than the hole mobility, resulting in dipole emission primarily at the HTL/EML interface. RMS surface roughness values were taken to be between 0 and 8 nm for the ETL, which were determined experimentally by AFM measurements of NBPhen films deposited on silicon.[4] The broadband emission spectrum of Alq_3 with a peak at 526 nm was applied to the results to account for the wavelength-dependent emission strength. An anisotropic distribution of dipole orientations (30:1 horizontal to vertically oriented dipoles) was used to achieve the best correspondence with experimental results.[5,6] This value of anisotropy was kept constant for all computational experiments.

Angle-Resolved Electroluminescence

Microcavity devices exhibit several characteristic features in their off-normal emission patterns. First, the peak wavelength of the waveguided mode inside the cavity exhibits a cosine dependence on the propagating angle, which manifests as a blue-shift in the resonant wavelength at higher viewing angles.[7] Thus a microcavity tuned to 550 nm (green) in the normal direction may appear blue when viewed from the side.[4] Additionally, the difference in boundary conditions for the TE and TM modes within the metallic cavity result in a swallow-tail splitting of the propagation modes at high angle (>40°). The lower-energy branch corresponds to the TM mode while the TE mode forms the higher-energy branch.[4,7]

Stacking multiple microcavities on top of each other was not found to fundamentally alter these behaviors, in agreement with simulations. Supplementary Figures 1 and 2 show the angle-resolved electroluminescence spectrum (ARES) of the devices produced for this paper, which show the presence of both angular dispersion and polarization splitting. Each resonant peak exhibits a separate angular dispersion and splitting, confirming the presence of discrete waveguide modes in the MDC structure. This leads to the potential for overlap of the polarization branches of adjacent photonic states, which was observed in the N = 2 ARES spectrum at roughly 60°. The data suggest the possibility of differences in the dispersion experienced by different photonic peaks, visible in the difference in polarization splitting in Fig. 2c.

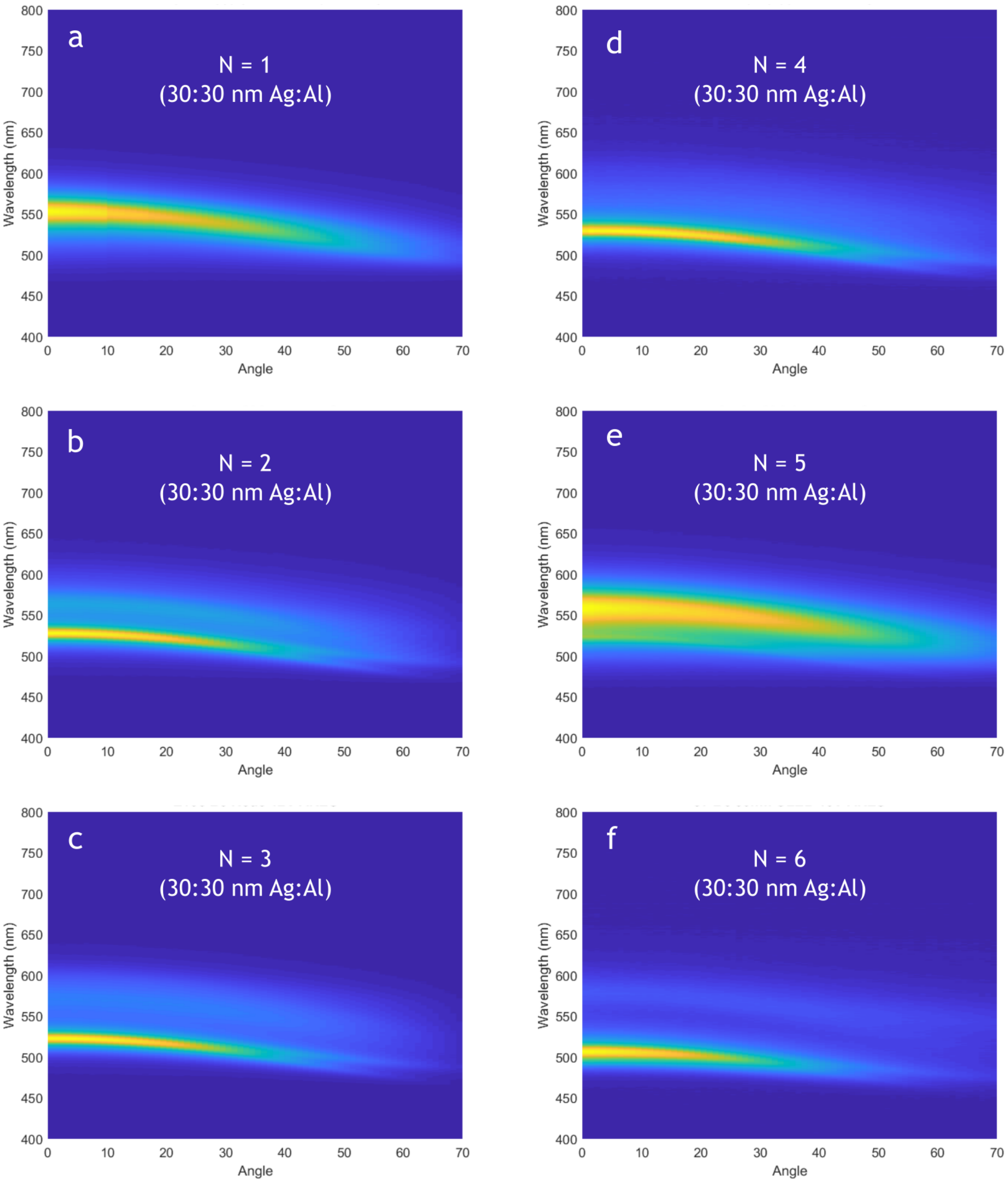

Supplementary Figure 1. ARES of the buildup experiment devices shown in Fig. 2. ARES dispersion maps of devices with **a** $N = 1$, **b** $N = 2$, **c** $N = 3$, **d** $N = 4$, **e** $N = 5$, and **f** $N = 6$ stacked microcavities. All mirrors are 30 nm thick.

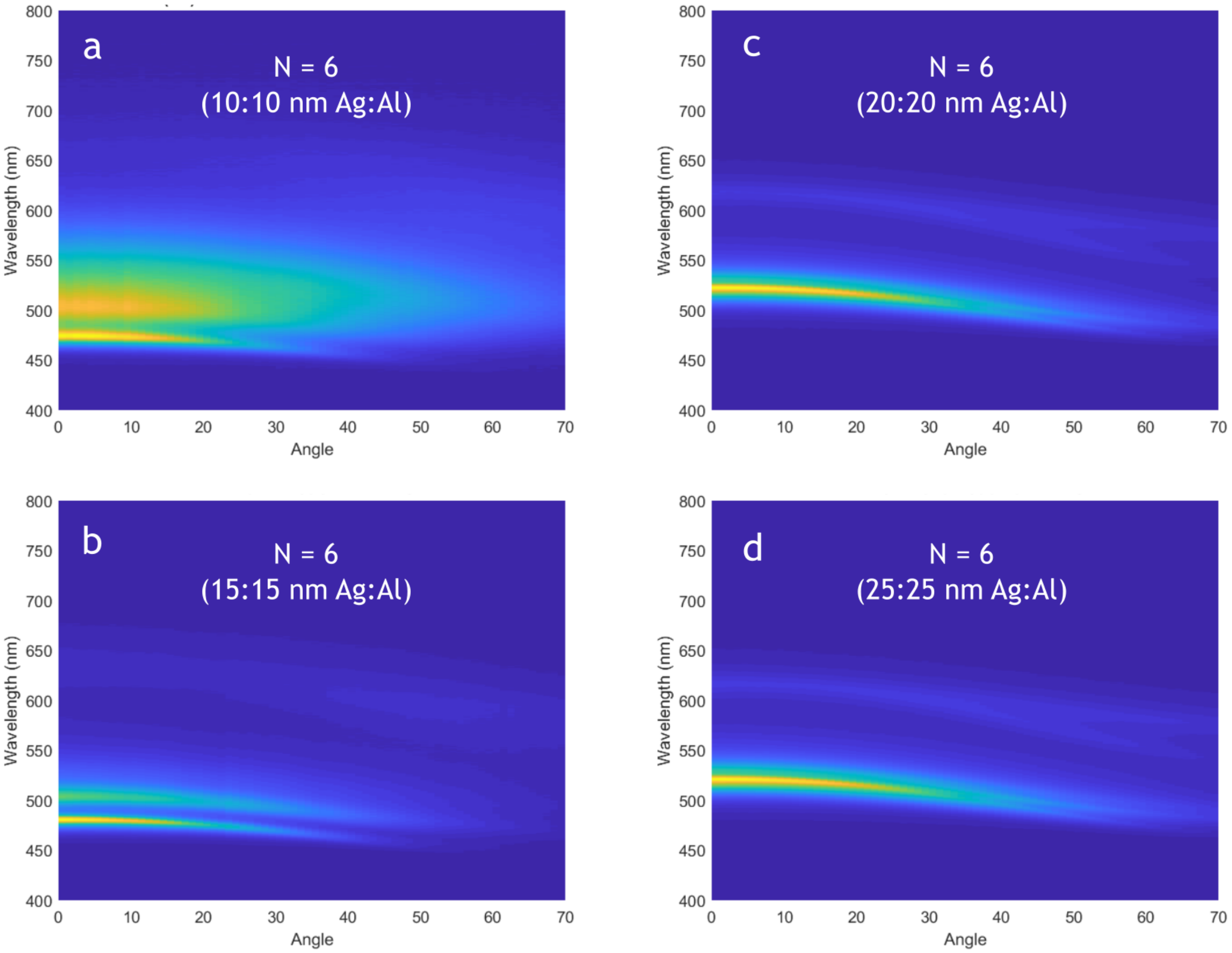

Supplementary Figure 2. ARES of the density of states experiment devices shown in Fig. 3. ARES dispersion maps of $N = 6$ stacked microcavity devices using mirrors of thickness **a** 10 nm, **b** 15 nm, **c** 20 nm, and **d** 25 nm.

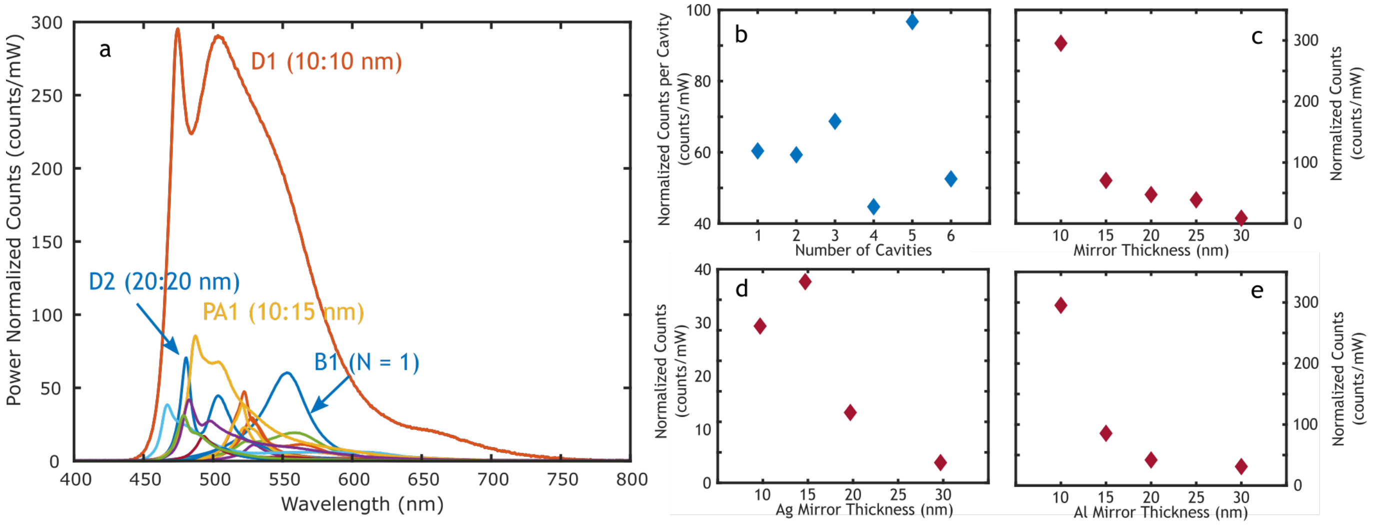

Supplementary Figure 3. Luminance of the device structures. a Comparison of normalized counts for the buildup, density of states and Peierls distortion devices. The four highest efficiency emitters are labeled. **b** Normalized counts per cavity for the buildup experiment of Figure 2a. This normalization ensures equivalent power input per cavity due to distribution over the stack. **c** Normalized counts for the density of states devices of Figure 3a, showing the trend in emission as the mirror thickness is reduced. **d** Normalized counts for the Ag Peierls devices of Figure 4a, showing trend in the emission as the silver mirror is reduced. **e** Normalized counts for the Al Peierls devices of Figure 4b, showing trend in the emission as the aluminum mirror is reduced.

Luminance Comparison

A comparison of the emission intensity from the devices was performed using the raw counts recorded by the spectrometer under equivalent measurement conditions of aperture diameter and integration time. The raw count data was normalized using the recorded power input to the device. This provides a rough measure of comparison between the devices of the paper. We generally observed an increase in efficiency as the mirror thickness was reduced, as can be seen in Supplementary Figure 3c-e. Thinner Al mirrors appear to dramatically increase the device efficiency, consistent with Al having a higher extinction coefficient than Ag. The efficiency per cavity was found to remain roughly steady as the number of cavities was increased during the buildup experiment, as shown in Fig. 3b, with the possibility of differences between the even and odd N devices which have Al and Ag top mirrors respectively. The overall best-performing device was the 10:10 (Ag:Al) device of Fig. 3, which showed almost three times higher emission per watt of input power compared with the next highest device.

Shift of the Band Center

The schematic of Figure 1a was presented as a model for the formation of photonic bands, with a key feature being the splitting of the isolated energy states into bands centered on those states. However, in Figure 3 it is shown that the band center shifts as the mirrors are made thinner. This contradiction is the result of the isolated energy states being a function of the mirror thickness d. Equation 1 indicates that the resonant energy of the states in a metallic cavity depends on the penetration depth ϕ. For the case where $d < \phi$, this equation no longer applies and we can roughly replace ϕ with the mirror thickness d. This results in a larger (lower energy) resonant wavelength. Crystals formed from these unit cells inherit this red shift, as shown in Supplementary Figure 4. This results in the observed discrepancy between the 30 nm single cavity state and the measured band center in panels (b)-(d) and demonstrates the effect predicted by the schematic in Figure 1a as the band shifts in response to the underlying states of the unit cell.

A second feature that can be seen in Supplementary Figure 4 is that the single cavity energies calculated with the appropriate mirror thickness represent the center of the band more accurately than simply finding the energy between the outer-most states of the band (dashed lines in Supplementary Figure 4). The center of the band using the outer-most states lies at a slightly higher energy than the equivalent single-cavity state and encroaches on the lower sub-band. This feature was observed in all of the simulations, with a deviation that increased as the mirrors were made thinner. At 10 nm, as shown in Supplementary Figure 4d, the band center nearly coincides with one of the peaks. This discrepancy is due to dispersion in the silver mirrors that distorts the band structure. The increase in bandwidth as the mirrors are made thinner results in a higher deviation between the center measured using the outer-most states and the single-cavity state.

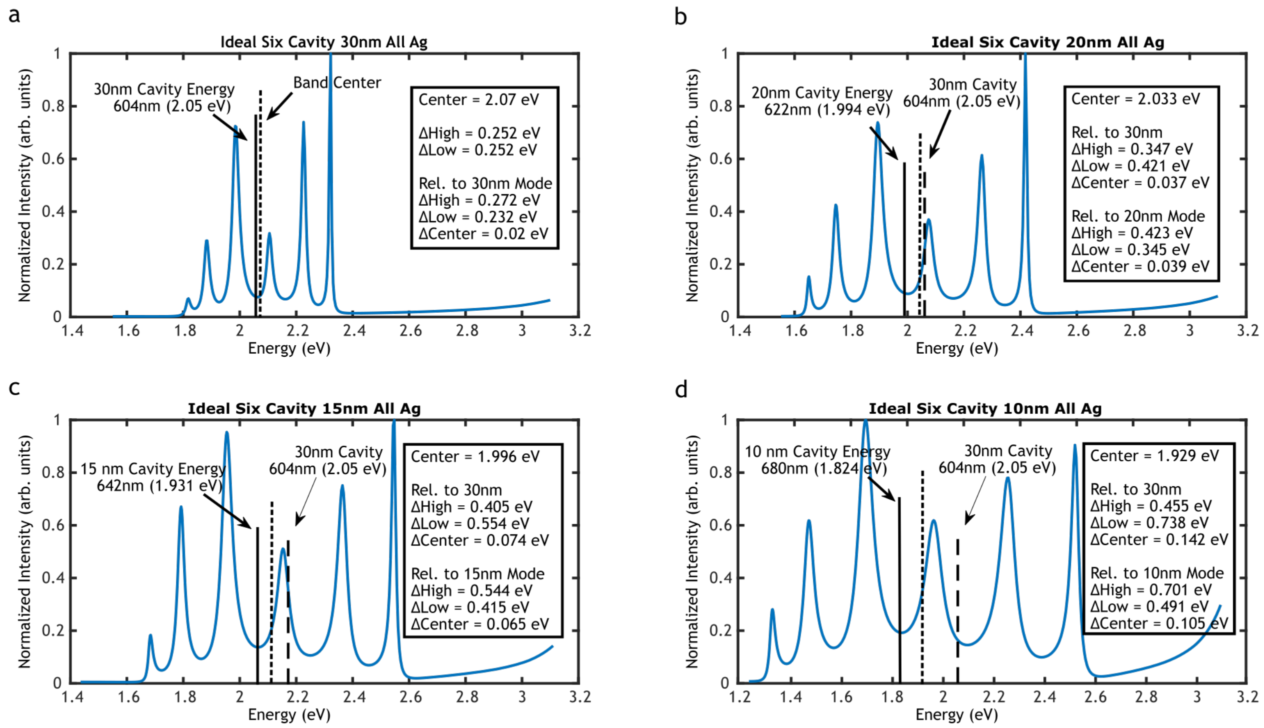

Supplementary Figure 4. Computational experiment showing the shift in band center. a-d Simulated emission spectra for idealized stacked microcavities consisting of only silver mirrors and 115 nm organic layers of index of refraction n = 1.8. The center of each band was calculated as the energy between the highest and lowest energy peaks, indicated by a dashed line.

Alternatively, it is possible to define the center based on the inner-most states rather than the outer-most. Crystals consisting of single-cavity unit cells have evenly spaced energy states, as in the Ag-only crystal shown in Supplementary Figure 4. In this case, the inner-most states provide an accurate indication of the position of the band center. In other cases, such as in the presence of a band gap due to alternating mirrors or defects in the crystal as in the experimental devices of this paper, the inner-most states may not be the best measure and the structure may be better characterized by its composite bands separately.

Emergence of Sub-bands

In the main text of this article, the emergence of the upper and lower sub-bands was described using a unit cell where the silver mirror thickness was taken to zero, leaving a double-length unit cell bounded by aluminum mirrors. The realities of device fabrication and performance at this time preclude an experimental investigation of the photonic band emergence from such a unit cell as the silver anodes cannot be made arbitrarily small. Using the transfer matrix simulation described above, however, it is possible to shed light on this phenomenon.

Eliminating the silver mirror from the unit cell employed in our devices (Figure 1d) leaves a single cell of length 230 nm bounded by aluminum mirrors. The fundamental and first harmonic modes of this unit cell are located at 472 nm and 981 nm, respectively, as shown in Supplementary Figure 5 on the left. The introduction of a second and third unit cell to the stack results in a splitting of both of these modes into additional hybridized states as in Figure 1a. These hybridized states form the upper and lower sub-bands observed in our experimental devices. The thickness of the aluminum mirrors controls the separation between the hybridized states, with thinner mirrors resulting in more broadly spaced states.

The silver mirror can be gradually added back into the unit cell to show the emergence of the experimentally observed bands from these idealized states. As shown in the central section of Supplementary Figure 5, adding even 1 nm of silver results in a dramatic change in the gap between the upper and lower sub-bands due to the combined effects of perturbation bringing fundamental mode higher in energy and the additional optical pathlength reducing the energy of the first harmonic. Thicker silver mirrors produce diminishing reductions in the photonic band gap and, to a lesser extent, the sub-band bandwidths. The states at 30:30 nm reflect those observed experimentally, with all states lying within the visible range. Going further, the mirrors can be made arbitrarily thick to observe the asymptotic behavior of the photonic bands. Continuing to increase the mirror thickness beyond 30 nm results in a further reduction in the band gap and sub-band bandwidths. The states approach the

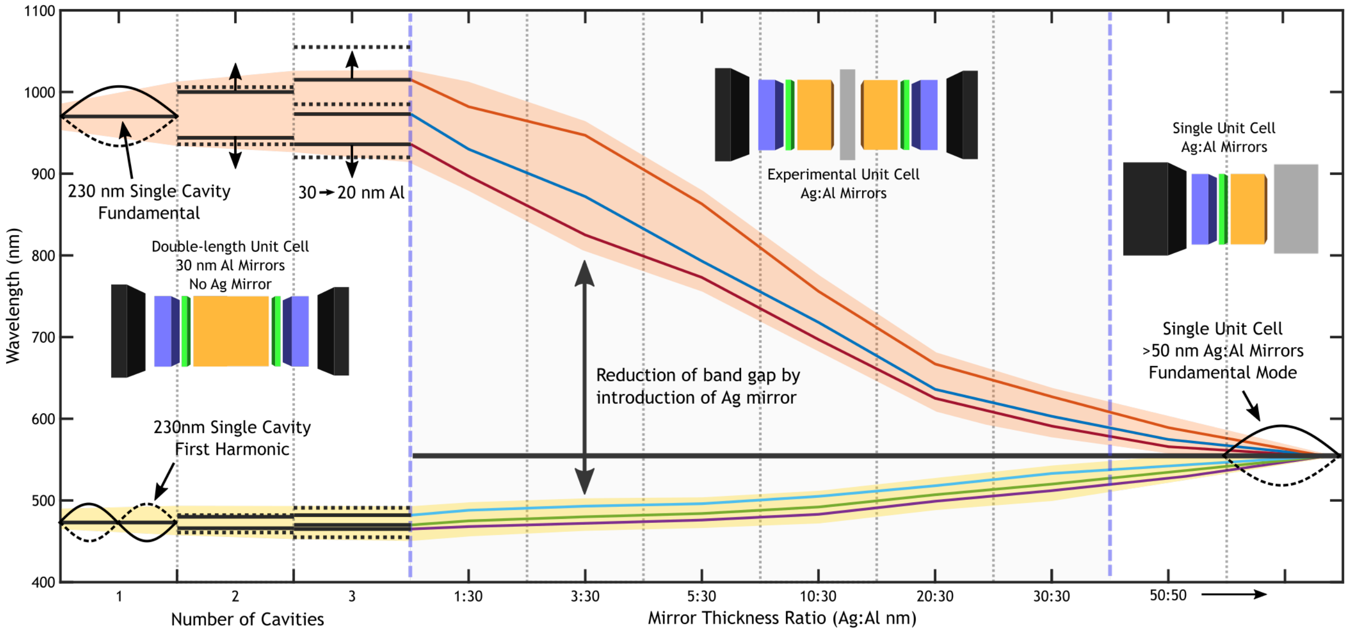

Supplementary Figure 5. Simulated data showing the emergence of the upper and lower sub-bands. The fundamental and first harmonic modes of a double-length unit cell bounded by Al mirrors are shown on the left. The first harmonic mode is higher energy than the fundamental of the Ag:Al asymmetric single cavity shown on the right due to the larger extinction coefficient in Al. Changing the Al mirror thickness affects the bandwidth of the resulting states as N increases (dashed states). Introducing a silver mirror in the center of the double-length unit cell reduces the gap between the sub-bands through perturbation of the fundamental mode (center). Both sub-bands converge to the fundamental mode of a single unit cell bounded by one Ag and one Al mirror (right).

fundamental mode of a single 115 nm unit cell bounded by one Ag and one Al mirror of arbitrary thickness. The energy of this fundamental mode differs from that of the single 230 nm unit cell bounded by only Al mirrors, with a wavelength of 562 nm compared to 472 nm due to the increased penetration depth of the silver mirror. Accordingly, the behavior of the MDC at intermediate mirror thickness depends on the relative energies of the single and double unit cell states (left-most and right-most states of Supplementary Figure 5). Replacing the silver dividing mirror with a different material or otherwise altering the composite unit cells would alter the asymptote of the sub-band energies and could result in dramatically different behavior at intermediate mirror thickness.

Modulation of the Peierls Band Gap

A series of computational experiments were conducted to expand on the discussion in the main text of the roles of the silver and aluminum mirrors in modulating the Peierls band gap and bandwidth. As discussed above, the upper and lower sub-bands are derived from the underlying states of the double-length unit cell defined by the aluminum mirrors in the absence of any silver mirror. While the separation between the sub-bands is fixed by the organic layer thickness, the separation of states within the sub-bands is controlled by the aluminum mirror thickness (see Supplementary Figure 5, left). Accordingly, Supplementary Figure 6a shows the increase in the bandwidth of the upper and lower sub-bands as the aluminum mirror thickness is reduced as the states approach those of the extended cavity as in Figure 1a on the right. This behavior is more apparent at large sub-band separation, a feature which guided the choice of 10 nm Ag mirrors rather than 30 nm for the data presented in Figure 4b.

The variation of the silver mirror thickness results in a modulation of the photonic band gap between the upper and lower sub-bands. As shown in Supplementary Figure 6b, this increase in the band gap does not result in a large change in the bandwidth of the sub-bands. Most of the change that can be observed, particularly with very thin mirrors as in Supplementary Figure 5, can be attributed to dispersion effects in the metal layers as the states spread in energy. The response of the photonic band gap is due to the perturbation of the modes of the Al-Al double unit cell by the internal Ag mirror. This perturbation is primarily experienced by the fundamental mode due to the overlap of the silver mirror with the antinode of the state.

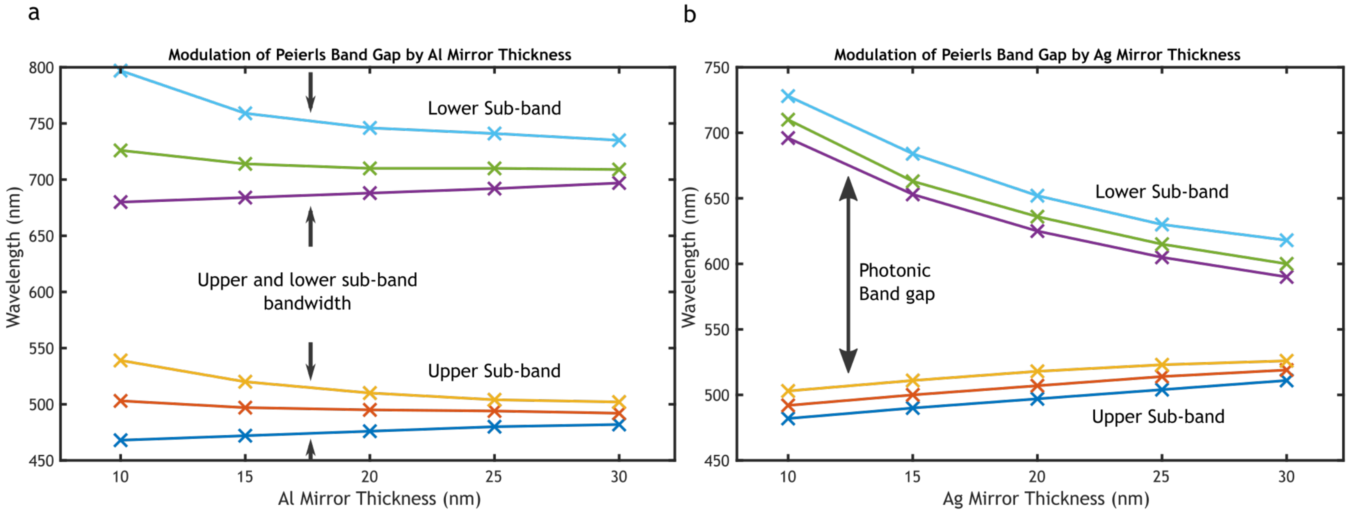

Supplementary Figure 6. Computational experiments showing the behavior of the Peierls band gap. a Simulated peak position data showing the effect of changing the aluminum mirror thickness in a six-cavity stack with 10 nm Ag mirrors. The thickness of the aluminum mirrors primarily controls the sub-band bandwidth. **b** Simulated peak position data showing the effect of changing the silver mirror thickness in a six-cavity stack with 30 nm Al mirrors. The thickness of the silver mirror primarily controls band gap between the upper and lower sub-bands.

References

1. Benisty, H., Stanley, R. & Mayer, M. Method of source terms for dipole emission modification in modes of arbitrary planar structures. *J. Opt. Soc. Am. A* **15**, 1192–1201, DOI: 10.1364/JOSAA.15.001192 (1998).
2. Mitsas, C. L. & Siapkas, D. I. Generalized matrix method for analysis of coherent and incoherent reflectance and transmittance of multilayer structures with rough surfaces, interfaces, and finite substrates. *Appl. Opt.* **34**, 1678–1683, DOI: 10.1364/AO.34.001678 (1995).
3. Pettersson, L. A. A., Roman, L. S. & Inganäs, O. Modeling photocurrent action spectra of photovoltaic devices based on organic thin films. *J. Appl. Phys.* **86**, 487–496 (1999).
4. Dahal, E., Allemeier, D., Isenhart, B., Cianciulli, K. & White, M. S. Characterization of higher harmonic modes in fabry–pérot microcavity organic light emitting diodes. *Sci. Reports* **11**, 1–12 (2021).
5. Lee, T. *et al.* The molecular origin of anisotropic emission in an organic light-emitting diode. *Nano Lett.* **17**, 6464–6468, DOI: 10.1021/acs.nanolett.7b03528 (2017). PMID: 28891653, https://doi.org/10.1021/acs.nanolett.7b03528.
6. Halls, M. D. & Schlegel, H. B. Molecular orbital study of the first excited state of the oled material tris(8-hydroxyquinoline)aluminum(iii). *Chem. Mater.* **13**, 2632–2640, DOI: 10.1021/cm010121d (2001). https://doi.org/10.1021/cm010121d.
7. Benisty, H., De Neve, H. & Weisbuch, C. Impact of planar microcavity effects on light extraction-part i: basic concepts and analytical trends. *IEEE J. Quantum Electron.* **34**, 1612–1631, DOI: 10.1109/3.709578 (1998).
