# Supplementary material for: Emergence and control of photonic band structure in stacked OLED microcavities
Source: Nat Commun. 2021 Oct 20;12:6111. doi: 10.1038/s41467-021-26440-3 (PMC8528838; doi:10.1038/s41467-021-26440-3)
Supplement: Supplementary file 3 — Description of Additional Supplementary Files [file 41467_2021_26440_MOESM3_ESM.pdf]

Description of Additional Supplementary Files

File name: Supplementary Data 1

Description: Code and all required data and documentation to conduct simulation of the stacked microcavity OLED devices.
