# Supplementary material for: Emergence and control of photonic band structure in stacked OLED microcavities
Source: Nat Commun. 2021 Oct 20;12:6111. doi: 10.1038/s41467-021-26440-3 (PMC8528838; doi:10.1038/s41467-021-26440-3)
Supplement: Supplementary file 4 — Supplementary Data 1 [file 41467_2021_26440_MOESM4_ESM.zip › OLED Simulation v2-1/OLED Simulation/Materials Data/Materials Database/doc/Dispersion formulas.docx]

**Dispersion formulas**

***1:*** ***Sellmeier (preferred)***

$$n^{2}-1=C_{1}+\frac{C_{2}\lambda^{2}}{\lambda^{2}-C_{3}^{2}}+\frac{C_{4}\lambda^{2}}{\lambda^{2}-C_{5}^{2}}+\frac{C_{6}\lambda^{2}}{\lambda^{2}-C_{7}^{2}}+\frac{C_{8}\lambda^{2}}{\lambda^{2}-C_{9}^{2}}+\frac{C_{10}\lambda^{2}}{\lambda^{2}-C_{11}^{2}}+\frac{C_{12}\lambda^{2}}{\lambda^{2}-C_{13}^{2}}+\frac{C_{14}\lambda^{2}}{\lambda^{2}-C_{15}^{2}}+\frac{C_{16}\lambda^{2}}{\lambda^{2}-C_{17}^{2}}$$

***2:*** ***Sellmeier-2***

$$n^{2}-1=C_{1}+\frac{C_{2}\lambda^{2}}{\lambda^{2}-C_{3}}+\frac{C_{4}\lambda^{2}}{\lambda^{2}-C_{5}}+\frac{C_{6}\lambda^{2}}{\lambda^{2}-C_{7}}+\frac{C_{8}\lambda^{2}}{\lambda^{2}-C_{9}}+\frac{C_{10}\lambda^{2}}{\lambda^{2}-C_{11}}+\frac{C_{12}\lambda^{2}}{\lambda^{2}-C_{13}}+\frac{C_{14}\lambda^{2}}{\lambda^{2}-C_{15}}+\frac{C_{16}\lambda^{2}}{\lambda^{2}-C_{17}}$$

***3: Polynomial***

$$n^{2}=C_{1}+C_{2}\lambda^{C_{3}}+C_{4}\lambda^{C_{5}}+C_{6}\lambda^{C_{7}}+C_{8}\lambda^{C_{9}}+C_{10}\lambda^{C_{11}}+C_{12}\lambda^{C_{13}}+C_{14}\lambda^{C_{15}}+C_{16}\lambda^{C_{17}}$$

***4: RefractiveIndex.INFO***

$$n^{2}=C_{1}+\frac{C_{2}\lambda^{C_{3}}}{\lambda^{2}-{C_{4}}^{C_{5}}}+\frac{C_{6}\lambda^{C_{7}}}{\lambda^{2}-{C_{8}}^{C_{9}}}+C_{10}\lambda^{C_{11}}+C_{12}\lambda^{C_{13}}+C_{14}\lambda^{C_{15}}+C_{16}\lambda^{C_{17}}$$

***5: Cauchy***

$$n=C_{1}+C_{2}\lambda^{C_{3}}+C_{4}\lambda^{C_{5}}+C_{6}\lambda^{C_{7}}+C_{8}\lambda^{C_{9}}+C_{10}\lambda^{C_{11}}$$

***6: Gases***

$$n-1=C_{1}+\frac{C_{2}}{C_{3}-\lambda^{-2}}+\frac{C_{4}}{C_{5}-\lambda^{-2}}+\frac{C_{6}}{C_{7}-\lambda^{-2}}+\frac{C_{8}}{C_{9}-\lambda^{-2}}+\frac{C_{10}}{C_{11}-\lambda^{-2}}$$

***7: Herzberger***

$$n=C_{1}+\frac{C_{2}}{\lambda^{2}-0.028}+C_{3}\left( \frac{1}{\lambda^{2}-0.028} \right)^{2}+C_{4}\lambda^{2}+C_{5}\lambda^{4}+C_{6}\lambda^{6}$$

***8: Retro***

$$\frac{n^{2}-1}{n^{2}+2}=C_{1}+\frac{C_{2}\lambda^{2}}{\lambda^{2}-C_{3}}+C_{4}\lambda^{2}$$

***9: Exotic***

$$n^{2}=C_{1}+\frac{C_{2}}{\lambda^{2}-C_{3}}+\frac{C_{4}(\lambda-C_{5})}{{(\lambda-C_{5})}^{2}+C_{6}}$$
