# Supplementary material for: Emergence and control of photonic band structure in stacked OLED microcavities
Source: Nat Commun. 2021 Oct 20;12:6111. doi: 10.1038/s41467-021-26440-3 (PMC8528838; doi:10.1038/s41467-021-26440-3)
Supplement: Supplementary file 4 — Supplementary Data 1 [file 41467_2021_26440_MOESM4_ESM.zip › OLED Simulation v2-1/OLED Simulation/Materials Data/Materials Database/doc/Dispersion formulas.pdf]

Dispersion formulas

1: Sellmeier (preferred)

$$n^2-1=C_1+\frac{C_2\lambda^2}{\lambda^2-C_3^2}+\frac{C_4\lambda^2}{\lambda^2-C_5^2}+\frac{C_6\lambda^2}{\lambda^2-C_7^2}+\frac{C_8\lambda^2}{\lambda^2-C_9^2}+\frac{C_{10}\lambda^2}{\lambda^2-C_{11}^2}+\frac{C_{12}\lambda^2}{\lambda^2-C_{13}^2}+\frac{C_{14}\lambda^2}{\lambda^2-C_{15}^2}+\frac{C_{16}\lambda^2}{\lambda^2-C_{17}^2}$$

2: Sellmeier-2

$$n^2-1=C_1+\frac{C_2\lambda^2}{\lambda^2-C_3}+\frac{C_4\lambda^2}{\lambda^2-C_5}+\frac{C_6\lambda^2}{\lambda^2-C_7}+\frac{C_8\lambda^2}{\lambda^2-C_9}+\frac{C_{10}\lambda^2}{\lambda^2-C_{11}}+\frac{C_{12}\lambda^2}{\lambda^2-C_{13}}+\frac{C_{14}\lambda^2}{\lambda^2-C_{15}}+\frac{C_{16}\lambda^2}{\lambda^2-C_{17}}$$

3: Polynomial

$$n^2=C_1+C_2\lambda^{C_3}+C_4\lambda^{C_5}+C_6\lambda^{C_7}+C_8\lambda^{C_9}+C_{10}\lambda^{C_{11}}+C_{12}\lambda^{C_{13}}+C_{14}\lambda^{C_{15}}+C_{16}\lambda^{C_{17}}$$

4: RefractiveIndex.INFO

$$n^2=C_1+\frac{C_2\lambda^{C_3}}{\lambda^2-C_4{}^{C_5}}+\frac{C_6\lambda^{C_7}}{\lambda^2-C_8{}^{C_9}}+C_{10}\lambda^{C_{11}}+C_{12}\lambda^{C_{13}}+C_{14}\lambda^{C_{15}}+C_{16}\lambda^{C_{17}}$$

5: Cauchy

$$n=C_1+C_2\lambda^{C_3}+C_4\lambda^{C_5}+C_6\lambda^{C_7}+C_8\lambda^{C_9}+C_{10}\lambda^{C_{11}}$$

6: Gases

$$n-1=C_1+\frac{C_2}{C_3-\lambda^{-2}}+\frac{C_4}{C_5-\lambda^{-2}}+\frac{C_6}{C_7-\lambda^{-2}}+\frac{C_8}{C_9-\lambda^{-2}}+\frac{C_{10}}{C_{11}-\lambda^{-2}}$$

7: Herzberger

$$n=C_1+\frac{C_2}{\lambda^2-0.028}+C_3\left(\frac{1}{\lambda^2-0.028}\right)^2+C_4\lambda^2+C_5\lambda^4+C_6\lambda^6$$

8: Retro

$$\frac{n^2-1}{n^2+2}=C_1+\frac{C_2\lambda^2}{\lambda^2-C_3}+C_4\lambda^2$$

9: Exotic

$$n^2=C_1+\frac{C_2}{\lambda^2-C_3}+\frac{C_4(\lambda-C_5)}{(\lambda-C_5)^2+C_6}$$

RefractiveIndex.INFO
2014-06-29
