# Supplementary material for: Emergence and control of photonic band structure in stacked OLED microcavities
Source: Nat Commun. 2021 Oct 20;12:6111. doi: 10.1038/s41467-021-26440-3 (PMC8528838; doi:10.1038/s41467-021-26440-3)
Supplement: Supplementary file 4 — Supplementary Data 1 [file 41467_2021_26440_MOESM4_ESM.zip › OLED Simulation v2-1/OLED Simulation/Materials Data/Materials Database/info/organic/glycerol.html]

# Glycerol, C3H8O3

## Chemical formula

C3H5(OH)3

## Other names

- Glycerin
- Glycerine
- Propane-1,2,3-triol
- 1,2,3-Propanetriol
- 1,2,3-Trihydroxypropane
- Glyceritol
- Glycyl alcohol

## External links

- Glycerol - Wikipedia
- Glycerol - NIST Chemistry WebBook
