# Supplementary material for: Emergence and control of photonic band structure in stacked OLED microcavities
Source: Nat Commun. 2021 Oct 20;12:6111. doi: 10.1038/s41467-021-26440-3 (PMC8528838; doi:10.1038/s41467-021-26440-3)
Supplement: Supplementary file 4 — Supplementary Data 1 [file 41467_2021_26440_MOESM4_ESM.zip › OLED Simulation v2-1/OLED Simulation/Materials Data/Materials Database/info/organic/poly(methyl methacrylate).html]

# Poly(methyl methacrylate), (C5H8O2)n

## Other names

- Poly(methyl methacrylate)
- PMMA
- Acrylic glass

## Trademarks

- Plexiglas
- Vitroflex
- Limacryl
- R-Cast
- Per-Clax
- Perspex
- Plazcryl
- Acrylex
- Acrylite
- Acrylplast
- Altuglas
- Polycast
- Oroglass
- Lucite

## External links

- Poly(methyl methacrylate) - Wikipedia
- PMMA/acrylic plastics: Complete guide - Omnexus
