# Supplementary material for: Emergence and control of photonic band structure in stacked OLED microcavities
Source: Nat Commun. 2021 Oct 20;12:6111. doi: 10.1038/s41467-021-26440-3 (PMC8528838; doi:10.1038/s41467-021-26440-3)
Supplement: Supplementary file 4 — Supplementary Data 1 [file 41467_2021_26440_MOESM4_ESM.zip › OLED Simulation v2-1/OLED Simulation/Materials Data/Materials Database/info/organic/propylene glycol.html]

# Propylene glycol, C3H8O2

## Other names

- Propane-1,2-diol
- α-Propylene glycol
- 1,2-Propanediol
- 1,2-Dihydroxypropane
- Methyl ethyl glycol (MEG)
- Methylethylene glycol

## External links

- Propylene glycol - Wikipedia
- Propylene glycol - NIST Chemistry WebBook
