# Supplementary material for: Emergence and control of photonic band structure in stacked OLED microcavities
Source: Nat Commun. 2021 Oct 20;12:6111. doi: 10.1038/s41467-021-26440-3 (PMC8528838; doi:10.1038/s41467-021-26440-3)
Supplement: Supplementary file 4 — Supplementary Data 1 [file 41467_2021_26440_MOESM4_ESM.zip › OLED Simulation v2-1/OLED Simulation/Materials Data/Materials Database/info/organic/dimethyl sulfoxide.html]

# Dimethyl sulfoxide, C2H6OS (DMSO, Me2SO)

## Chemical formula

(CH3)2SO

## Other names

- Methanesulfinylmethane (substitutive)
- Dimethyl(oxido)sulfur (additive)
- Methylsulfinylmethane
- Methyl sulfoxide

## External links

- Dimethyl sulfoxide - Wikipedia
- Dimethyl sulfoxide - PubChem
- Dimethyl sulfoxide - NIST Chemistry WebBook
