# Supplementary material for: Emergence and control of photonic band structure in stacked OLED microcavities
Source: Nat Commun. 2021 Oct 20;12:6111. doi: 10.1038/s41467-021-26440-3 (PMC8528838; doi:10.1038/s41467-021-26440-3)
Supplement: Supplementary file 4 — Supplementary Data 1 [file 41467_2021_26440_MOESM4_ESM.zip › OLED Simulation v2-1/OLED Simulation/Materials Data/Materials Database/info/organic/ethyl acetate.html]

# Ethyl acetate, C4H8O2 (EtOAc, EA)

## Chemical formula

CH3-COO-CH2-CH3

## Other names

- Ethyl ethanoate
- Acetic ester
- Acetic ether
- Ethyl ester
- Acetic acid

## External links

- Ethyl acetate - Wikipedia
- Ethyl acetate - PubChem
- Ethyl acetate - NIST Chemistry WebBook
