# Supplementary material for: Emergence and control of photonic band structure in stacked OLED microcavities
Source: Nat Commun. 2021 Oct 20;12:6111. doi: 10.1038/s41467-021-26440-3 (PMC8528838; doi:10.1038/s41467-021-26440-3)
Supplement: Supplementary file 4 — Supplementary Data 1 [file 41467_2021_26440_MOESM4_ESM.zip › OLED Simulation v2-1/OLED Simulation/Materials Data/Materials Database/info/organic/chloroform.html]

# Chloroform, CHCl3

## Other names

- Trichloromethane
- Formyl trichloride
- Methane trichloride
- Methyl trichloride
- Methenyl trichloride
- TCM
- Freon 20
- Refrigerant-20
- R-20
- UN 1888

## External links

- Chloroform - Wikipedia
- Methane, trichloro- - NIST Chemistry WebBook
