# Supplementary material for: Emergence and control of photonic band structure in stacked OLED microcavities
Source: Nat Commun. 2021 Oct 20;12:6111. doi: 10.1038/s41467-021-26440-3 (PMC8528838; doi:10.1038/s41467-021-26440-3)
Supplement: Supplementary file 4 — Supplementary Data 1 [file 41467_2021_26440_MOESM4_ESM.zip › OLED Simulation v2-1/OLED Simulation/Materials Data/Materials Database/info/organic/acetone.html]

# Acetone, C3H6O

## Chemical formula

CH3COCH3

## Other names

- Propanone
- Dimethyl ketone
- 2-Propanone
- Propan-2-one
- Dimethylformaldehyde
- β-Ketopropane
- DMK

## External links

- Acetone - Wikipedia
- Acetone - NIST Chemistry WebBook
