# Supplementary material for: Emergence and control of photonic band structure in stacked OLED microcavities
Source: Nat Commun. 2021 Oct 20;12:6111. doi: 10.1038/s41467-021-26440-3 (PMC8528838; doi:10.1038/s41467-021-26440-3)
Supplement: Supplementary file 4 — Supplementary Data 1 [file 41467_2021_26440_MOESM4_ESM.zip › OLED Simulation v2-1/OLED Simulation/Materials Data/Materials Database/info/organic/methyl salicylate.html]

# Methyl salicylate, C8H8O3

## Chemical formula

C6H4(HO)COOCH3

## Other names

- Salicylic acid methyl ester
- Oil of wintergreen
- Betula oil
- Methyl-2-Hydroxybenzoate

## External links

- Methyl salicylate - Wikipedia
- Methyl salicylate - NIST Chemistry WebBook
