# Supplementary material for: Emergence and control of photonic band structure in stacked OLED microcavities
Source: Nat Commun. 2021 Oct 20;12:6111. doi: 10.1038/s41467-021-26440-3 (PMC8528838; doi:10.1038/s41467-021-26440-3)
Supplement: Supplementary file 4 — Supplementary Data 1 [file 41467_2021_26440_MOESM4_ESM.zip › OLED Simulation v2-1/OLED Simulation/Materials Data/Materials Database/info/organic/ethylene-vinyl acetate.html]

# Ethylene-vinyl acetate, (C2H4)n(C4H6O2)m (EVA)

## Other names

- Poly(ethylene-vinyl acetate) (PEVA)
- Poly(ethylene-co-vinyl acetate)
- Polyethylene-vinyl acetate copolymer

## Trademarks

- EVASKY

## External links

- Ethylene-vinyl acetate - Wikipedia
- What's EVASKY - Bridgestone
