# Supplementary material for: Emergence and control of photonic band structure in stacked OLED microcavities
Source: Nat Commun. 2021 Oct 20;12:6111. doi: 10.1038/s41467-021-26440-3 (PMC8528838; doi:10.1038/s41467-021-26440-3)
Supplement: Supplementary file 4 — Supplementary Data 1 [file 41467_2021_26440_MOESM4_ESM.zip › OLED Simulation v2-1/OLED Simulation/Materials Data/Materials Database/info/organic/polydimethylsiloxane.html]

# Polydimethylsiloxane, (C2H6OSi)n

## Other names

- PDMS
- Poly(dimethylsiloxane)
- Dimethicone
- Dimethylpolysiloxane

## Food additive code

- E900

## Trademarks

- SF-96 (Momentive)
- RTV 615 (Momentive)
- Sylgard 184 (Dow)

## External links

- Polydimethylsiloxane - Wikipedia
