# Supplementary material for: Emergence and control of photonic band structure in stacked OLED microcavities
Source: Nat Commun. 2021 Oct 20;12:6111. doi: 10.1038/s41467-021-26440-3 (PMC8528838; doi:10.1038/s41467-021-26440-3)
Supplement: Supplementary file 4 — Supplementary Data 1 [file 41467_2021_26440_MOESM4_ESM.zip › OLED Simulation v2-1/OLED Simulation/Materials Data/Materials Database/info/organic/acetonitrile.html]

# Acetonitrile, C2H3N

## Chemical formula

CH3CN

## Other names

- Cyanomethane
- Ethanenitrile
- Ethyl nitrile
- Methanecarbonitrile
- Methyl cyanide

## External links

- Acetonitrile - Wikipedia
- Acetonitrile - NIST Chemistry WebBook
