# Supplementary material for: Emergence and control of photonic band structure in stacked OLED microcavities
Source: Nat Commun. 2021 Oct 20;12:6111. doi: 10.1038/s41467-021-26440-3 (PMC8528838; doi:10.1038/s41467-021-26440-3)
Supplement: Supplementary file 4 — Supplementary Data 1 [file 41467_2021_26440_MOESM4_ESM.zip › OLED Simulation v2-1/OLED Simulation/Materials Data/Materials Database/info/organic/dimethyl methylphosphonate.html]

# Dimethyl methylphosphonate, C3H9O3P

## Other names

- DMMP
- Methylphosphonic acid dimethyl ester
- dimethoxymethyl phosphine oxide
- Dimethyl methanephosphonate
- Dimethyl methyl phosphonate
- Fran TF 2000
- Fyron DMMP
- Metaran
- NSC 62240
- O,O-dimethyl methylphosphonate
- Reoflam DMMP

## External links

- Dimethyl methylphosphonate - Wikipedia
- Dimethyl methylphosphonate - NIST Chemistry WebBook
