# Supplementary material for: Emergence and control of photonic band structure in stacked OLED microcavities
Source: Nat Commun. 2021 Oct 20;12:6111. doi: 10.1038/s41467-021-26440-3 (PMC8528838; doi:10.1038/s41467-021-26440-3)
Supplement: Supplementary file 4 — Supplementary Data 1 [file 41467_2021_26440_MOESM4_ESM.zip › OLED Simulation v2-1/OLED Simulation/Materials Data/Materials Database/info/organic/poly(N-isopropylacrylamide).html]

# Poly(N-isopropylacrylamide), (C6H11NO)n

## Abbreviations

- PNIPAM
- PNIPA
- PNIPAAm
- NIPA
- PNIPAA
- PNIPAm

## External links

- Poly(N-isopropylacrylamide) - Wikipedia
