# Supplementary material for: Emergence and control of photonic band structure in stacked OLED microcavities
Source: Nat Commun. 2021 Oct 20;12:6111. doi: 10.1038/s41467-021-26440-3 (PMC8528838; doi:10.1038/s41467-021-26440-3)
Supplement: Supplementary file 4 — Supplementary Data 1 [file 41467_2021_26440_MOESM4_ESM.zip › OLED Simulation v2-1/OLED Simulation/Materials Data/Materials Database/info/organic/polylactic acid.html]

# Polylactic acid, (C3H4O2)n

## Other names

- Poly(lactic acid)
- Polylactide
- PLA

## Variations

- PLA: Polylactide
- PDLA: Poly-D-lactide
- PLLA: Poly-L-lactide
- PDLLA: Poly-DL-lactide

## External links

- Polylactic acid - Wikipedia
