# Supplementary material for: Emergence and control of photonic band structure in stacked OLED microcavities
Source: Nat Commun. 2021 Oct 20;12:6111. doi: 10.1038/s41467-021-26440-3 (PMC8528838; doi:10.1038/s41467-021-26440-3)
Supplement: Supplementary file 4 — Supplementary Data 1 [file 41467_2021_26440_MOESM4_ESM.zip › OLED Simulation v2-1/OLED Simulation/Materials Data/Materials Database/info/organic/styrene.html]

# Styrene, C8H8

## Chemical formula

C6H5CH=CH2

## Other names

- Phenylethene
- Ethenylbenzene
- Vinyl benzene
- Cinnamene
- Styrol
- Phenylethene
- Diarex HF 77
- Styrolene
- Styropol
- Vinylbenzene
- Phenylethylene

## External links

- Styrene - Wikipedia
- Styrene - NIST Chemistry WebBook
