# Supplementary material for: Emergence and control of photonic band structure in stacked OLED microcavities
Source: Nat Commun. 2021 Oct 20;12:6111. doi: 10.1038/s41467-021-26440-3 (PMC8528838; doi:10.1038/s41467-021-26440-3)
Supplement: Supplementary file 4 — Supplementary Data 1 [file 41467_2021_26440_MOESM4_ESM.zip › OLED Simulation v2-1/OLED Simulation/Materials Data/Materials Database/info/organic/propanol.html]

# Propanol, C3H8O

## Chemical formula

- 1-Propanol: CH3CH2CH2OH
- Isopropanol: (CH3)2CHOH

## Other names

| 1-Propanol | Isopropanol |
| --- | --- |
| - Propan-1-ol - n-Propyl alcohol - n-Propanol - n-PrOH - Ethylcarbinol - 1-Hydroxypropane - Propanol - Propionic alcohol - Propionyl alcohol - Propionylol - Propyl alcohol - Propylic alcohol - Propylol | - Isopropyl alcohol - 2-Propanol - Propan-2-ol - Isopropyl alcohol - Rubbing alcohol - Sec-propyl alcohol - s-Propanol - iPrOH - IPA |

## External links

- 1-Propanol - Wikipedia
- 1-Propanol - NIST Chemistry WebBook
- Isopropyl alcohol - Wikipedia
- Isopropyl alcohol - NIST Chemistry WebBook
