# Supplementary material for: Emergence and control of photonic band structure in stacked OLED microcavities
Source: Nat Commun. 2021 Oct 20;12:6111. doi: 10.1038/s41467-021-26440-3 (PMC8528838; doi:10.1038/s41467-021-26440-3)
Supplement: Supplementary file 4 — Supplementary Data 1 [file 41467_2021_26440_MOESM4_ESM.zip › OLED Simulation v2-1/OLED Simulation/Materials Data/Materials Database/info/organic/polyvinylpyrrolidone.html]

# Polyvinylpyrrolidone, (C6H9NO)n

## Other names

- PVP
- Povidone
- Copovidone
- PVPP
- Crospovidone
- Polyvidone
- PNVP
- Poly[1-(2-oxo-1-pyrrolidinyl)ethylen]
- 1-Ethenyl-2-pyrrolidon homopolymer
- 1-Vinyl-2-pyrrolidinon-Polymere

## External links

- Polyvinylpyrrolidone - Wikipedia
