# Supplementary material for: Emergence and control of photonic band structure in stacked OLED microcavities
Source: Nat Commun. 2021 Oct 20;12:6111. doi: 10.1038/s41467-021-26440-3 (PMC8528838; doi:10.1038/s41467-021-26440-3)
Supplement: Supplementary file 4 — Supplementary Data 1 [file 41467_2021_26440_MOESM4_ESM.zip › OLED Simulation v2-1/OLED Simulation/Materials Data/Materials Database/info/organic/pentanol.html]

# Pentanol (Amyl alcohol), C5H12O

## Chemical formula

- Normal amyl alcohol: CH3CH2CH2CH2OH or C5H11OH
- Isoamyl alcohol: (CH3)2CHCH2CH2OH

## Other names

| Normal amyl alcohol | Isoamyl alcohol |
| --- | --- |
| - 1-Pentanol - n-Pentanol - Pentan-1-ol | - 3-Methyl-1-Butanol - 3-Methylbutan-1-ol - Isopentyl alcohol - Isopentanol - Isobutylcarbinol |

## External links

- Amyl alcohol - Wikipedia
- 1-Pentanol - Wikipedia
- 1-Pentanol - NIST Chemistry WebBook
- Isoamyl alcohol - Wikipedia
- 1-Butanol, 3-methyl- - NIST Chemistry WebBook
