# Supplementary material for: Emergence and control of photonic band structure in stacked OLED microcavities
Source: Nat Commun. 2021 Oct 20;12:6111. doi: 10.1038/s41467-021-26440-3 (PMC8528838; doi:10.1038/s41467-021-26440-3)
Supplement: Supplementary file 4 — Supplementary Data 1 [file 41467_2021_26440_MOESM4_ESM.zip › OLED Simulation v2-1/OLED Simulation/Materials Data/Materials Database/info/organic/butanol.html]

# Butanol, C4H10O

## Chemical formula

- n-Butanol: CH3(CH2)3OH or C4H9OH
- sec-Butanol: CH3CH(OH)CH2CH3
- Isobutanol: (CH3)2CHCH2OH
- tert-Butanol: (CH3)3COH

## Other names

Butyl alcohol

| n-Butanol | sec-Butanol | Isobutanol | tert-Butanol |
| --- | --- | --- | --- |
| - Butan-1-ol - Butalcohol - Butanol - 1-Butanol - Butyl alcohol - Butyl hydrate - Butylic alcohol - Butyralcohol - Butyric alcohol - Butyryl alcohol - Hydroxybutane - Propylcarbinol | - Butan-2-ol - 2-Butanol - sec-Butyl alcohol - 2-Butyl alcohol | - 2-Methylpropan-1-ol - Isobutyl alcohol - IBA - 2-Methyl-1-propanol - 1-Propanol, 2-methyl- - 2-Methylpropyl alcohol | - tert-Butyl alcohol (TBA) - 2-Methylpropan-2-ol - 2-Methyl-2-propanol (2M2P) - 2-Propanol, 2-methyl- |

## External links

- Butanol - Wikipedia
- n-Butanol - Wikipedia
- 1-Butanol - NIST Chemistry WebBook
- 2-Butanol - Wikipedia
- 2-Butanol - NIST Chemistry WebBook
- Isobutanol - Wikipedia
- 1-Propanol, 2-methyl- - NIST Chemistry WebBook
- tert-Butyl alcohol - Wikipedia
- 2-Propanol, 2-methyl- - NIST Chemistry WebBook
