# Supplementary material for: Emergence and control of photonic band structure in stacked OLED microcavities
Source: Nat Commun. 2021 Oct 20;12:6111. doi: 10.1038/s41467-021-26440-3 (PMC8528838; doi:10.1038/s41467-021-26440-3)
Supplement: Supplementary file 4 — Supplementary Data 1 [file 41467_2021_26440_MOESM4_ESM.zip › OLED Simulation v2-1/OLED Simulation/Materials Data/Materials Database/info/organic/potassium hydrogen phthalate.html]

# Potassium hydrogen phthalate, C8H5KO4

## Other names

- Hydrogen potassium phthalate
- Phthalic acid potassium salt
- Potassium biphthalate
- Potassium acid phthalate
- 1,2-Benzenedicarboxylic acid
- Monopotassium salt
- KHP
- KHPh

## External links

- Potassium hydrogen phthalate - Wikipedia
