# Supplementary material for: Emergence and control of photonic band structure in stacked OLED microcavities
Source: Nat Commun. 2021 Oct 20;12:6111. doi: 10.1038/s41467-021-26440-3 (PMC8528838; doi:10.1038/s41467-021-26440-3)
Supplement: Supplementary file 4 — Supplementary Data 1 [file 41467_2021_26440_MOESM4_ESM.zip › OLED Simulation v2-1/OLED Simulation/Materials Data/Materials Database/info/organic/pentanediol.html]

# Pentanediol, C5H12O2

## Isomers

- 1,5-Pentanediol - most common isomer
- 2,4-Pentanediol
- 1,2-Pentanediol

## Chemical formula

1,5-Pentanediol: HOCH2CH2CH2CH2CH2OH

## Other names

1,5-Pentanediol:

- Pentane-1,5-diol
- Pentylene glycol
- Pentamethylene glycol
- 1,5-Dihydroxypentane

## External links

- 1,5-Pentanediol - Wikipedia
- 1,5-Pentanediol - NIST Chemistry WebBook
- 2,4-Pentanediol - NIST Chemistry WebBook
- 1,2-Pentanediol - NIST Chemistry WebBook
