# Supplementary material for: Emergence and control of photonic band structure in stacked OLED microcavities
Source: Nat Commun. 2021 Oct 20;12:6111. doi: 10.1038/s41467-021-26440-3 (PMC8528838; doi:10.1038/s41467-021-26440-3)
Supplement: Supplementary file 4 — Supplementary Data 1 [file 41467_2021_26440_MOESM4_ESM.zip › OLED Simulation v2-1/OLED Simulation/Materials Data/Materials Database/info/organic/dioxane.html]

# Dioxane, C4H8O2

## Isomers

- 1,4-Dioxane (p-Dioxane) - most common isomer
- 1,3-Dioxane (m-Dioxane)
- 1,2-Dioxane (o-Dioxane)

## Other names

1,4-Dioxane:

- p-Dioxane
- Dioxane
- 4-Dioxacyclohexane
- [1,4]Dioxane
- [6]-Crown-2

## External links

- 1,4-Dioxane - Wikipedia
- 1,4-Dioxane - NIST Chemistry WebBook
- 1,3-Dioxane - NIST Chemistry WebBook
