# Supplementary material for: Emergence and control of photonic band structure in stacked OLED microcavities
Source: Nat Commun. 2021 Oct 20;12:6111. doi: 10.1038/s41467-021-26440-3 (PMC8528838; doi:10.1038/s41467-021-26440-3)
Supplement: Supplementary file 4 — Supplementary Data 1 [file 41467_2021_26440_MOESM4_ESM.zip › OLED Simulation v2-1/OLED Simulation/Materials Data/Materials Database/info/organic/carbon tetrachloride.html]

# Carbon chloride, CCl4

## Other names

- Benziform
- Carbon chloride
- Methane tetrachloride
- Perchloromethane
- Carbon tet
- Benzinoform
- Tetraform
- Tetrasol
- Freon 10
- Halon 104

## External links

- Carbon tetrachloride - Wikipedia
- Carbon tetrachloride - NIST Chemistry WebBook
