# Supplementary material for: Emergence and control of photonic band structure in stacked OLED microcavities
Source: Nat Commun. 2021 Oct 20;12:6111. doi: 10.1038/s41467-021-26440-3 (PMC8528838; doi:10.1038/s41467-021-26440-3)
Supplement: Supplementary file 4 — Supplementary Data 1 [file 41467_2021_26440_MOESM4_ESM.zip › OLED Simulation v2-1/OLED Simulation/Materials Data/Materials Database/info/organic/ethyl salicylate.html]

# Ethyl salicylate, C9H10O3

## Chemical formula

2-(HO)C6H4CO2C2H5

## Other names

- Ethyl 2-hydroxybenzoate
- Benzoic acid, 2-hydroxy-, ethyl ester

## External links

- Ethyl salicylate - Wikipedia
- Benzoic acid, 2-hydroxy-, ethyl ester - NIST Chemistry WebBook
