# Supplementary material for: Emergence and control of photonic band structure in stacked OLED microcavities
Source: Nat Commun. 2021 Oct 20;12:6111. doi: 10.1038/s41467-021-26440-3 (PMC8528838; doi:10.1038/s41467-021-26440-3)
Supplement: Supplementary file 4 — Supplementary Data 1 [file 41467_2021_26440_MOESM4_ESM.zip › OLED Simulation v2-1/OLED Simulation/Materials Data/Materials Database/info/other/ZrO2-Y2O3.html]

# Yttria-stabilized zirconia (YSZ), ZrO2-Y2O3

## Other names

- Zirconium dioxide - Yttrium oxide

Zirconium dioxide other name:

- Zirconia

Yttrium oxide other names:

- Yttria
- Yttrium(III) oxide

## External links

- Yttria-stabilized zirconia - Wikipedia
- Zirconium dioxide - Wikipedia
- Yttrium(III) oxide - Wikipedia
