# Supplementary material for: Emergence and control of photonic band structure in stacked OLED microcavities
Source: Nat Commun. 2021 Oct 20;12:6111. doi: 10.1038/s41467-021-26440-3 (PMC8528838; doi:10.1038/s41467-021-26440-3)
Supplement: Supplementary file 4 — Supplementary Data 1 [file 41467_2021_26440_MOESM4_ESM.zip › OLED Simulation v2-1/OLED Simulation/Materials Data/Materials Database/info/other/PTB7-PC71BM.html]

# PTB7:PC71BM

## Applications

- Solar cells

## Chemical composition

PTB7: (C41H53FO4S4)n

## Other names (PTB7)

- Poly({4,8-bis[(2-ethylhexyl)oxy]benzo[1,2-b:4,5-b′]dithiophene-2,6-diyl}{3-fluoro-2-[(2-ethylhexyl)carbonyl]thieno[3,4-b]thiophenediyl})

## Other names (PC71BM)

- [6,6]-phenyl-C71-butyric acid methyl ester

## External links

- PTB7 - Sigma Aldrich
