# Supplementary material for: Emergence and control of photonic band structure in stacked OLED microcavities
Source: Nat Commun. 2021 Oct 20;12:6111. doi: 10.1038/s41467-021-26440-3 (PMC8528838; doi:10.1038/s41467-021-26440-3)
Supplement: Supplementary file 4 — Supplementary Data 1 [file 41467_2021_26440_MOESM4_ESM.zip › OLED Simulation v2-1/OLED Simulation/Materials Data/Materials Database/info/other/anti-reflective coatings.html]

# Index-matching materials

An antireflective or anti-reflection coating is a type of optical coating applied to the surface of lenses and other optical elements to reduce reflection.

## External links

- Anti-reflective coating - Wikipedia
- Anti-reflective coatings - Encyclopedia of laser physics and technology
