# Supplementary material for: Emergence and control of photonic band structure in stacked OLED microcavities
Source: Nat Commun. 2021 Oct 20;12:6111. doi: 10.1038/s41467-021-26440-3 (PMC8528838; doi:10.1038/s41467-021-26440-3)
Supplement: Supplementary file 4 — Supplementary Data 1 [file 41467_2021_26440_MOESM4_ESM.zip › OLED Simulation v2-1/OLED Simulation/Materials Data/Materials Database/info/other/P3HT-PC61BM.html]

# P3HT:PC61BM

## Applications

- Solar cells

## Chemical composition

P3HT: (C41H53FO4S4)n
PC61BM: C72H14O2

## Other names (P3HT)

- Poly(3-hexylthiophene-2,5-diyl)
- Plexcore OS 2100

## Other names (PC61BM)

- [6,6]-phenyl-C61-butyric acid methyl ester

## External links

- P3HT - Sigma Aldrich
- PC61BM - Wikipedia
