# Supplementary material for: Emergence and control of photonic band structure in stacked OLED microcavities
Source: Nat Commun. 2021 Oct 20;12:6111. doi: 10.1038/s41467-021-26440-3 (PMC8528838; doi:10.1038/s41467-021-26440-3)
Supplement: Supplementary file 4 — Supplementary Data 1 [file 41467_2021_26440_MOESM4_ESM.zip › OLED Simulation v2-1/OLED Simulation/Materials Data/Materials Database/info/other/Ni-Fe.html]

# Nickel-Iron alloys, Ni-Fe

## Other names and abbreviations

- nickel–iron alloy
- FeNi
- NiFe

## Popular alloys

- Invar (~36% Ni, low thermal expansion)
- Permalloy (~80% Ni, highly magnetic)

## External links

- Iron–nickel alloy - Wikipedia
- Invar - Wikipedia
- Permalloy - Wikipedia
- Permalloy-80 - ESPI
