# Supplementary material for: Emergence and control of photonic band structure in stacked OLED microcavities
Source: Nat Commun. 2021 Oct 20;12:6111. doi: 10.1038/s41467-021-26440-3 (PMC8528838; doi:10.1038/s41467-021-26440-3)
Supplement: Supplementary file 4 — Supplementary Data 1 [file 41467_2021_26440_MOESM4_ESM.zip › OLED Simulation v2-1/OLED Simulation/Materials Data/Materials Database/info/other/C6H4S4-C12H4N4.html]

# Tetrathiafulvalene:tetracyanoquinodimethane (TTF:TCNQ), C6H4S4:C12H4N4

## External links

- Tetracyanoquinodimethane - Wikipedia
- Tetrathiafulvalene - Wikipedia
