# Supplementary material for: Emergence and control of photonic band structure in stacked OLED microcavities
Source: Nat Commun. 2021 Oct 20;12:6111. doi: 10.1038/s41467-021-26440-3 (PMC8528838; doi:10.1038/s41467-021-26440-3)
Supplement: Supplementary file 4 — Supplementary Data 1 [file 41467_2021_26440_MOESM4_ESM.zip › OLED Simulation v2-1/OLED Simulation/Materials Data/Materials Database/info/other/HfO2-Y2O3.html]

# Hafnium oxide - Yttrium oxide, HfO2-Y2O3

## Other names

HfO2 other names:

- Hafnia- Hafnium dioxide
  - Hafnium(IV) oxide

Y2O3 other names:

- Yttria
- Yttrium(III) oxide

## External links

- Hafnium(IV) oxide - Wikipedia
- Yttrium(III) oxide - Wikipedia
