# Supplementary material for: Emergence and control of photonic band structure in stacked OLED microcavities
Source: Nat Commun. 2021 Oct 20;12:6111. doi: 10.1038/s41467-021-26440-3 (PMC8528838; doi:10.1038/s41467-021-26440-3)
Supplement: Supplementary file 4 — Supplementary Data 1 [file 41467_2021_26440_MOESM4_ESM.zip › OLED Simulation v2-1/OLED Simulation/Materials Data/Materials Database/info/other/(C8H8)n-(C3H3N)m.html]

# Styrene-acrylonitrile (SAN), (C8H8)n-(C3H3N)m

## Other names

- Styrene-acrylonitrile resin
- Styrene-acrylonitrile copolymer

## External links

- Styrene-acrylonitrile resin - Wikipedia
- Styrene-acrylonitrile copolymer - Encyclopædia Britannica
