# Supplementary material for: Emergence and control of photonic band structure in stacked OLED microcavities
Source: Nat Commun. 2021 Oct 20;12:6111. doi: 10.1038/s41467-021-26440-3 (PMC8528838; doi:10.1038/s41467-021-26440-3)
Supplement: Supplementary file 4 — Supplementary Data 1 [file 41467_2021_26440_MOESM4_ESM.zip › OLED Simulation v2-1/OLED Simulation/Materials Data/Materials Database/info/glass/fused silica.html]

# Fused silica

Fused silica (or fused quartz, or quartz glass) is a type of glass containing primarily silica (SiO2) in amorphous (non-crystalline) form.

## External links

- Fused quartz - Wikipedia
- IR Grade Fused Silica - ICL
- UV Grade Fused Silica - ICL
