# Supplementary material for: Emergence and control of photonic band structure in stacked OLED microcavities
Source: Nat Commun. 2021 Oct 20;12:6111. doi: 10.1038/s41467-021-26440-3 (PMC8528838; doi:10.1038/s41467-021-26440-3)
Supplement: Supplementary file 4 — Supplementary Data 1 [file 41467_2021_26440_MOESM4_ESM.zip › OLED Simulation v2-1/OLED Simulation/Materials Data/Materials Database/info/glass/BAF10.html]

# BAF10 optical glass

BAF10 is a very popular optical glass. Most glass makers produce this or very similar glass under slightly different names.

## BAF10 and similar glasses produced by different makers

| Maker | Glass |
| --- | --- |
| Schott | N-BAF10 |
| Hikari | E-BAF10 |
| HOYA | BAF10 |
| Ohara | S-BAH10 |
| CDGM | H-ZBAF52 |
