# Supplementary material for: Emergence and control of photonic band structure in stacked OLED microcavities
Source: Nat Commun. 2021 Oct 20;12:6111. doi: 10.1038/s41467-021-26440-3 (PMC8528838; doi:10.1038/s41467-021-26440-3)
Supplement: Supplementary file 4 — Supplementary Data 1 [file 41467_2021_26440_MOESM4_ESM.zip › OLED Simulation v2-1/OLED Simulation/Materials Data/Materials Database/info/glass/CORNING.html]

# Corning Inc.

Corning Incorporated is an American manufacturer of glass, ceramics, and related materials, primarily for industrial and scientific applications. Among other products, Corning has developed and is manufacruring the Gorilla glass: a specialized toughened alkali-aluminosilicate glass used in the displays of most of the modern smartphones.

## External links

- Corning | Materials Science Technology and Innovation
- Corning Inc. - Wikipedia
- Corning Museum of Glass
- Gorilla Glass - Wikipedia
