# Supplementary material for: Emergence and control of photonic band structure in stacked OLED microcavities
Source: Nat Commun. 2021 Oct 20;12:6111. doi: 10.1038/s41467-021-26440-3 (PMC8528838; doi:10.1038/s41467-021-26440-3)
Supplement: Supplementary file 4 — Supplementary Data 1 [file 41467_2021_26440_MOESM4_ESM.zip › OLED Simulation v2-1/OLED Simulation/Materials Data/Materials Database/info/glass/BK7.html]

# BK7 optical glass

BK7 is a very popular crown glass. Most glass makers produce this glass under slightly different names.

## Analogs of BK7 glass produced by different makers

| Maker | Glass |
| --- | --- |
| SCHOTT | N-BK7 |
| OHARA | S-BSL7 |
| HIKARI | J-BK7A |
| CDGM | H-K9L |
| HOYA | BSC7 |
| SUMITA | K-BK7 |
| LZOS | K8 |

## External links

- Crown glass - Wikipedia
