# Supplementary material for: Emergence and control of photonic band structure in stacked OLED microcavities
Source: Nat Commun. 2021 Oct 20;12:6111. doi: 10.1038/s41467-021-26440-3 (PMC8528838; doi:10.1038/s41467-021-26440-3)
Supplement: Supplementary file 4 — Supplementary Data 1 [file 41467_2021_26440_MOESM4_ESM.zip › OLED Simulation v2-1/OLED Simulation/Materials Data/Materials Database/info/glass/infrared glass.html]

# Infrared glass

Often, calcogenide glasses are used in the optical systems operating at infrared wavelengths ~1-14 µm (for instance, optical systems for thermal imaging and night vision)

## Infrared glass types/brands from different makers

- **AMTIR** (an acronym for "Amorphous Material Transmitting Infrared Radiation"): Amorphous Materials Inc. (AMI)
- **IG**: VITRON
- **IRG**: SCHOTT
- **BD**: LightPath
- **OPTIR**: Rochester Precision Optics (RPO)

## Cross-reference

| Composition | AMI | VITRON | SCHOTT | LightPath | RPO |
| --- | --- | --- | --- | --- | --- |
| Ge33As12Se55 | AMTIR-1 | IG 2 | IRG 22 |  | OPTIR-1\* |
| Ge30Sb13Se32Te25 |  | IG 3 | IRG 23\* |  |  |
| Ge10As40Se50 |  | IG 4 | IRG 24 |  |  |
| Ge28Sb12Se60 | AMTIR-3\* | IG 5 | IRG 25 | BD-2 | OPTIR-3\* |
| As40Se60 | AMTIR-2 | IG 6 | IRG 26 | BD6 |  |
| As40S60 | AMTIR-6 |  | IRG 27 |  |  |

(\*) Out of production

## External Links

- Infrared - Wikipedia
- Chalcogenide glass - Wikipedia
- AMTIR amorphous material transmitting IR radiation - Amorphous Materials
- Brief introduction: chalcogenide glasses - VITRON
- Infrared Chalcogenide Glasses - SCHOTT
- Optical resources and technical information - LightPath
