# Supplementary material for: Emergence and control of photonic band structure in stacked OLED microcavities
Source: Nat Commun. 2021 Oct 20;12:6111. doi: 10.1038/s41467-021-26440-3 (PMC8528838; doi:10.1038/s41467-021-26440-3)
Supplement: Supplementary file 4 — Supplementary Data 1 [file 41467_2021_26440_MOESM4_ESM.zip › OLED Simulation v2-1/OLED Simulation/Materials Data/Materials Database/info/glass/SF11.html]

# SF11 optical glass

SF11 is a popular optical glass. Most glass makers produce this or very similar glass under slightly different names.

## SF11 and similar glasses produced by different makers

| Maker | Glass |
| --- | --- |
| Schott | N-SF11 |
| Hikari | E-SF11 |
| Sumita | K-SFLD11 |
| CDGM | ZF13 |
