# Supplementary material for: Emergence and control of photonic band structure in stacked OLED microcavities
Source: Nat Commun. 2021 Oct 20;12:6111. doi: 10.1038/s41467-021-26440-3 (PMC8528838; doi:10.1038/s41467-021-26440-3)
Supplement: Supplementary file 4 — Supplementary Data 1 [file 41467_2021_26440_MOESM4_ESM.zip › OLED Simulation v2-1/OLED Simulation/Materials Data/Materials Database/info/glass/OHARA.html]

# Ohara Group

Ohara Group is an international group of companies with headquarters in Japan and subsidiaries in Japan (Ohara, Inc.), United States (Ohara Corporation), and Germany (Ohara, GmbH). Ohara manufactures optical and electronic products and also offers measurement services, in particular measurement of refractive indices of solids and liquids.

## External links

- Ohara, Inc.
- Ohara Corporation
- Ohara, GmbH
- Ohara glass catalog
