# Supplementary material for: Emergence and control of photonic band structure in stacked OLED microcavities
Source: Nat Commun. 2021 Oct 20;12:6111. doi: 10.1038/s41467-021-26440-3 (PMC8528838; doi:10.1038/s41467-021-26440-3)
Supplement: Supplementary file 4 — Supplementary Data 1 [file 41467_2021_26440_MOESM4_ESM.zip › OLED Simulation v2-1/OLED Simulation/Materials Data/Materials Database/info/main/Sc2O3.html]

# Scandium sesquioxide, Sc2O3

## Other names

- Scandia
- Scandium(III) oxide
- Discandium trioxide
- Scandium oxide

## External links

- Scandium(III) oxide - Wikipedia
- Discandium trioxide - WebElements
- Scandium oxide applications - Scandium Information Center
